# Treatment of Chronic Hepatitis C in a Patient Affected by Systemic Sclerosis

**DOI:** 10.1155/2009/475390

**Published:** 2009-11-17

**Authors:** Guido Poggi, Laura Villani, Federico Sottotetti, Barbara Tagliaferri, Benedetta Montagna, Alessio Amatu, Giovanni Bernardo

**Affiliations:** ^1^Department of Oncology, IRCCS Maugeri Foundation, via Maugeri 8, 27100 Pavia, Italy; ^2^Department of Pathology, IRCCS Maugeri Foundation, via Maugeri 8, 27100 Pavia, Italy

## Abstract

The currently recommended treatment for patients infected with hepatitis C virus (HCV) is pegilated interferon *α* (IFN *α*) plus ribavirin. Despite the numerous benefits of this therapy, there is an increasing concern regarding his tolerance. Among the most common side effects, interferon may trigger the onset or exacerbation of autoimmune diseases. When chronic hepatitis C coexists with an autoimmune disorder, it is not clear whether using interferon is better than avoiding it. We evaluated the disease state of a 55-year old female affected by sistemic sclerosis (SSc), during and after therapy with IFN*α* pegilated plus ribavirin for chronic HCV infection. We were worried about the potential worsening of the autoimmune disease during the therapy, but we were confident that we would give our patient a short course of peginterferon and ribavirin. A mild, asymptomatic worsening of lung SSc was observed during IFN administration, without life threatening symptoms. After 24 months follow up we observed the maintenance of the virological response and a good control of the rheumatological disease. Thus, in liver disease at high risk of progression and concomitant SSc, the antiviral therapy with IFN*α* is a feasible approach.

## 1. Introduction

The currently recommended treatment for patients infected with hepatitis C virus (HCV) is pegylated interferon *α* plus ribavirin [1].

Despite the numerous benefits of interferon-based therapies, there is an increasing concern regarding its tolerance. Around 12% of patients on therapy cease treatment due to adverse effects and 2% do so as a consequence of laboratory abnormalities [2]. Among the most common side effects, interferon may trigger the development of autoantibodies in about 50% of patients, or even the onset or exacerbation of autoimmune diseases in 2% of them. So when chronic hepatitis C (CHC) coexists, in the same patient, with an autoimmune disorder, it is not clear whether using interferon is better than avoiding it.

Several cases of Systemic sclerosis (SSc) developed simultaneously with the use of interferon *α* are described in the literature, suggesting its promoting role in the occurrence of the disease [3–5], but nothing is reported about the treatment of CHC with INF *α* in patients already affected by SSc. In this paper we describe the clinical course of a patient affected by SSc during and after the six-month peginterferon *α* and ribavirin therapy for CHC. Systemic sclerosis (SSc) is a systemic complex connective tissue disease characterized by the deposition of excessive amounts of normal extracellular matrix components, particularly collagen, in the skin, subcutaneous tissue, muscles, and internal organs. The etiology of SSc is unknown but oligoclonal T-cell expansion, suggestive of an antigen-driven process, and an increase in the production of fibrogenic cytokines play an important role in the development of this disease [6].

## 2. Case Report

A 55-year-old woman was seen at our hospital because of elevated alanine aminotrasferase (ALT) and asparatate aminotrasferase (AST). She had been diagnosed as having chronic hepatitis C virus infection approximately 10 years earlier, in 1996. The viral genotype was 2a/2c. ALT and AST values were in the normal range at diagnosis and remained normal throughout bi-annual followups. Liver biopsy gave a diagnosis of chronic hepatitis with minimal activity and absence of fibrosis (A1, F0, Metavir). No antiviral therapy was planned and the patient was advised to continue the monitoring of her liver enzymes.

Five years after this first evaluation the patient complained of bilateral Raynaud's phenomenon and morning stiffness. Laboratory tests showed the presence of antinuclear antibody (ANA) (1 : 640) with centromeric pattern. Cryoglobulins were not detectable in serum. A scleroderma pattern was observed by capillaroscopy while pulmonary function tests were normal, showing only a mild reduction in the diffusing capacity of carbon monoxide (DLCO, 60% predicted). No interstitial lung disease was found by high-resolution computed tomography, and echocardiography showed normal left ventricular systolic function. Bronchoalveolar lavage fluid was normal. According to clinical and laboratory findings the patient was diagnosed as having mild SSc with cutaneous and pulmonary involvement, without pulmonary hypertension. Over the next few years the Raynaud's phenomenon became somewhat more frequent and DLCO was slightly decreased.

Ten years after the diagnosis of hepatitis C virus infection, hepatic enzymes suddenly increased for the first time, and remained quite elevated during the following months (Table 1). We decided to perform a liver biopsy that gave a diagnosis of severe worsening of the chronic hepatitis compared to the previous biopsy, with marked viral activity and severe fibrosis (A3, F3, Metavir). No histological clues addressing the diagnosis of autoimmune hepatitis were found. Specifically lobular necrosis and moderate portal inflammation were the predominant aspects of necroinflammatory activity without evidence of liver cell rosettes or plasmocytic portal infiltration. Although hepatitis C shares some histological features with autoimmune hepatitis, such as portal lymphoid aggregates or lymphoid follicles with germinal centers, they were not found in our patient. Other routine laboratory analyses were normal except for ANA test that was positive (1 : 640) with centromeric pattern. No specific autoantibodies were detected, including anti-ENA, anti-dsDNA, antimitochondrial (AMA), antismooth muscle (SMA), and antiliver-kidney microsomes (LKMs), antineutrophil cytoplasmic antiobodies (ANCAs), antisoluble liver antigens, and antiliver-pancreas (LP) autoantibodies. HCV viral load was elevated (3.131.400 IU/mL). The calculated score for autoimmune hepatitis, according to the International Scoring System, indicated a low probability of disease (Table 2) [7]. After an exhaustive discussion with the patient about pros and cons of treating chronic viral infection in this peculiar setting of associated connective tissue disease, the patient decided to receive treatment. The therapy, consisting in subcutaneous weekly injections of 180 *μ*g of peginterferon alfa-2a (Pegasys, Hoffman-LaRoche) plus 800 mg daily Ribavirin (Copegus, Hoffman-LaRoche), was started in December 2005 with progressive improvement of the liver function tests and fast decline of the HCV viremia. Four weeks after the beginning of the therapy, serum transaminases normalized and HCV RNA level, as measured with the use of a qualitative polymerase-chain-reaction assay (Cobas Amplicor HCV Test, version 2.0; detection limit, 50 IU per milliliter), was undetectable and remained so up to the end of the treatment (24 weeks). During the therapy and during the following two years pulmonary functional tests, capillaroscopy, and rheumatologic evaluations were performed to detect any worsening of the associated connective tissue disease. The followup was uneventful with stabilization of Raynaud's phenomenon and no worsening of lung function. The patient's main clinical and laboratory findings, before at the end and after 24 months after the end of therapy, are summarized in Table 1.

## 3. Discussion

Clinical studies on IFN*α* in the pathogenesis of autoimmune diseases have given contradictory results, either its detrimental or its protective role being suggested.

IFN*α* is of potential benefit in reducing fibrosis [8] since it has been shown to inhibit skin collagen synthesis and fibroblast proliferation, in vitro [9]. IFN*α* is also able to reduce the transforming growth factor *β*1 (TGF*β*1) messenger RNA when used in the treatment of chronic liver disease [10]. All the interferons have also been reported to be beneficial in the treatment of a variety of fibrotic diseases, including scleroderma [11]. IFN*α* therefore may be an appropriate treatment to retard fibrosis in early scleroderma. Despite such evidence, the results of a randomized, double blind, placebo-controlled clinical trial failed to show any improvement of the outcome in patients with diffuse cutaneous scleroderma treated with interferon [8].

Other studies focused on the role of cytokines, particularly type I IFN*α* and IFN*β*, in the development and perpetuation of autoimmunity [12].

IFN*α*-based therapies have been used for the treatment of specific malignancies and for treating chronic viral hepatitis C and B [13]. The mechanism of its activity is likely based on the capacity of exogenous IFN*α* to promote cellular effector functions that are mediated by cytotoxic T lymphocytes and natural killer cells. Furthermore, exogenous cytokines play a role in modulating dendritic cells activation and in the priming of naïve T cells.

Autoimmunity is a fairly common complication of IFN-based treatments, as indicated by several case reports that describe the development of autoimmune thyroid diseases, diabetes, SLE, polymyositis, psoriasis, inflammatory arthritis, Sjogren's syndrome, and Crohn's disease [14], particularly in the absence of predicting factors such as the presence of autoantibodies before the beginning of the therapy. During IFN*α* treatment, more than 50% of subjects develop autoantibodies, but only 1-2% show an evident autoimmune pathology [15, 16].

Systemic sclerosis (SSc) is a connective tissue multisystem disease, whose pathogenesis involves autoimmune phenomena, alteration of microvasculature and massive deposits of collagen, and other matrix substances in the connective tissue. Dysregulation of humoral immunity in SSc is best represented by the variety of autoantibodies against nuclear and nucleolar components, spontaneously produced by SSc patients, some of which are highly disease specific. In addition, recent reports suggest that antitopoisomerase antibodies directly bind fibroblasts, and that their serum levels correlate with SSc disease activity [17]. Abnormalities in cellular immunity are apparent from the increased number of T cells that bear markers of activation in peripheral blood of SSc patients.

In the literature, we have found only three cases of SSc occurring in female patients during or after treatment with IFN*α*. Two of them were treated for chronic hepatitis C and one for chronic myelogenous leukemia. The association between IFN*α* therapy and appearance of SSc has been strongly demonstrated only in one of these patients (Table 3).

Conversely there are no reports in the literature on the clinical outcome of patients formerly affected by SSc who received IFNa for chronic viral hepatitis C.

In our case report we evaluated the disease state of a 55-year-old female affected by SSc, during and after antiviral therapy with IFN*α* pegilated plus ribavirin for chronic viral hepatitis C.

It is well known that a sudden flare up of serum transaminase, particularly in genotype 2 or 3 hepatitis C, leads to severe worsening of liver fibrosis. This also happened to our patient [18, 19], and prompted us to start an antiviral therapy. We were worried about the potential worsening of the autoimmune disease during therapy with interferon, but we were confident that we would give our patient a short course of peginterferon and ribavirin. Indeed, data published short time before had shown no difference in viral clearance between short and conventional therapies in genotype 2 or 3 chronic hepatitis [20]. Furthermore, IFN has been tested also in patients with rheumatic disease, showing controversial outcomes, as demonstrated by Nissen et al. in their 2005 study, in which patients treated with interferon *α* for HCV with rheumatological symptoms showed deterioration in 50% of cases, no changes in 33%, and amelioration in 17% [21]. More encouraging results were reported by Zuckerman et al. who demonstrated acomplete or partial response of arthritis related symptoms in 44% of 25 HCV arthritis patients treated with interferon *α*_The authors concluded that interferon *α* may lead to_substantial clinical improvement of HCV related arthritis even without a complete biochemical or virological response [22].

We therefore decided to start an antiviral therapy with IFN*α* and ribavirin in our patient, despite her concomitant disease. During the therapy the patient underwent frequent followup visits. A mild, asymptomatic, and temporary worsening of lung SSc was observed at instrumental tests during IFN administration (decreased FVC%, decreased DLCO), in the absence of life threatening symptoms or severe discomfort. Black et al. pointed out that IFN*α* therapy might be deleterious in SSc patients since it exacerbates symptoms and precipitates lung deterioration through the upregulation of the intercellular type I adhesion molecule (ICAM1), a fibroblast adhesion molecule that is overexpressed in SSc fibroblasts and is believed to be important in mediating lymphocyte-fibroblast interactions [23]. In all the cases of SSc developed during treatment with inducing drugs, such as docetaxel, bleomycin, and other chemotherapeutic agents [24] the discontinuation of the treatment improved or reversed SSc symptoms. Also in our patient, at the end of the treatment, we observed a progressive normalization of lung function parameters, that soon returned to pretreatment values.

After 24 months of followup, we observed the maintenance of the virological response and a good control of the rheumatological disease. In conclusion, IFN*α* was effective in our patient, leading only to an asymptomatic mild worsening of SSc. Thus, in liver disease at high risk of progression and concomitant SSc, the antiviral therapy with IFN*α* is a feasible approach.

## Figures and Tables

**Table 1:** Disease activity parameters.

Variable	3 months before IFN therapy	2 months before IFN therapy	1 month before IFN therapy	1 week before IFN therapy	4th weeks of therapy	At the end of IFN	24 months after IFN
FVC (% predicted)				+0%		−10%	+0%
DLCO (% predicted)				−29%		−40%	−20%
White cells count × 10^9^				7		3.2	6.6
Red cells count × 10^12^				4.66		4.2	4.62
Hg (g/dl)				13.4		12	13
Platelet count × 10^9^				215		194	243
RCP				0.34		0.3	0.3
Creatinine (mg/dl)				0.89		0.6	0.7
ALT (IU/L)	857	437	237	241	36		
AST (IU/L)	424	291	156	168	36		
AP (IU/L)	422	344	291	275			
GGT (IU/L)	288	187	115	110			
Bilirubin (mg/dl)	0,4		0.4	0.3	0,3		
HCV-RNA viremia (IU/ml)				3131760	<50	<50	<50

**Table 2:** International scoring system for diagnosis of autoimmune hepatitis. This scoring system defines a “definitive autoimmune hepatitis” for values >+15 before treatment; “probable autoimmune hepatitis” is defined for values between 10, and 15 before treatment. Our patient presented total score +5 (very unlikely diagnosis of autoimmune hepatitis).

Parameter	Patient's results	Score	Parameter	Patient's results	Score
Gender	Female	+2	Hepatotoxic dugs exposure	No	+1
AP : AST ratio	<3.0	+2	Alcohol	<25 g/day	+2
*γ*-globulin level above-normal	1.0–1.5	+1	HLA DR3 o DR4	No	0
Autoantibodies	ANA >1 : 80	+3	Other autoimmune disease	yes	+2
Antimitochondrial Antibodies	Negative	0	Other markers	Negative	0
Viral markers	HCV RNA positive	−3	Histological features	None	−5

AP : AST ratio: ratio of alkaline phosphatase level to aspartate aminotransferase level; ANA: antinuclear antibodies.

**Table 3:** Case reports from literature.

Age	Gender	IFN	Pathology	Dose	Duration	Timing of disease appearance	Therapy	Trend	Author, year and references
66	Female	INF*α*con-1	Chronic viral hepatitis	18 MIU/daily for 2 weeks than 18 MIU/three times weekly	20 weeks	1 year after the end of IFN therapy	Tocopherol nicotinate	Partial relief with recurrent disease	Tahara et al. [5]
47	Female	INF*α*2b	Chronic viral hepatitis	3 MIU/three times weekly	Interrupted after 6 months	During therapy	Interruption of IFN therapy and prednisone 1 mg/kg/day	Limited form of systemic sclerosis	Solans et al. [3]
52	Female	INF*α*2a	Chronic myelogenous leukaemia	6 MIU/daily	25 months	During therapy	Loop diuretics, cyclophosphamide 100 mg/day, prostanoids, steroids	Partial improvement	Beretta et al. [4]
